# Visualizing risky situations induces a stronger neural response in brain areas associated with mental imagery and emotions than visualizing non-risky situations

**DOI:** 10.3389/fnhum.2023.1207364

**Published:** 2023-09-19

**Authors:** Tomasz Zaleskiewicz, Jakub Traczyk, Agata Sobkow, Kamil Fulawka, Alberto Megías-Robles

**Affiliations:** ^1^Faculty of Psychology in Wrocław, SWPS University of Social Sciences and Humanities, Wrocław, Poland; ^2^Facultad de Psicología, Universidad de Málaga, Málaga, Spain

**Keywords:** risk, mental imagery, emotions, neural correlates, neuroimaging

## Abstract

In an fMRI study, we tested the prediction that visualizing risky situations induces a stronger neural response in brain areas associated with mental imagery and emotions than visualizing non-risky and more positive situations. We assumed that processing mental images that allow for “trying-out” the future has greater adaptive importance for risky than non-risky situations, because the former can generate severe negative outcomes. We identified several brain regions that were activated when participants produced images of risky situations and these regions overlap with brain areas engaged in visual, speech, and movement imagery. We also found that producing images of risky situations, in contrast to non-risky situations, was associated with increased neural activation in the insular cortex and cerebellum–the regions involved, among other functions, in emotional processing. Finally, we observed an increased BOLD signal in the cingulate gyrus associated with reward-based decision making and monitoring of decision outcomes. In summary, risky situations increased neural activation in brain areas involved in mental imagery, emotional processing, and decision making. These findings imply that the evaluation of everyday risky situations may be driven by emotional responses that result from mental imagery.

## Introduction

Risk assessment is an important aspect of both professional and everyday decision making, where risky situations are defined as those that can generate both positive and negative outcomes for an individual (Yates and Stone, 1992). Although various expert domains (e.g., engineering or finance) require risk to be expressed in a numerical format (e.g., the size and the probability of potential outcomes), people often form their risk evaluations independently of quantitative parameters (Slovic, 1987; Sjoberg, 2000; Cousin and Siegrist, 2010; Renner et al., 2015; Weber, 2017). For example, subjective risk perception can be driven by negative emotions such as fear and anxiety (Sjoberg, 1998; Loewenstein et al., 2001; Slovic et al., 2005; Parrott, 2017; Zaleskiewicz and Traczyk, 2020). However, less is known about other psychological factors providing input to risk perception, and one such factor is mental imagery. In the present work, we proposed that mental imagery involved in the episodic simulation of future events may have the capacity to shape people’s perceptions of risk. In particular, we assumed that the exposure to risky (vs. non-risky and affectively more positive) situations would evoke stronger neural activation in brain regions associated with mental imagery and emotions that are typically elicited when people engage in producing and processing mental images (Blackwell, 2020). Observing such an effect on the level of brain activity would suggest that people use mental imagery more intensely when faced with risky than non-risky situations, because the psychological functions of mental imagery become especially important when decision makers are exposed to different threats.

### Mental imagery and its adaptive function in risk perception

According to a widely cited definition, “Mental imagery occurs when perceptual information is accessed from memory, giving rise to the experience of “seeing with the mind’s eye,” “hearing with the mind’s ear’ and so on…” (Kosslyn et al., 2001, p. 635). In this sense, mental imagery draws from memory (Kosslyn et al., 2001; Schacter et al., 2008) but refers to representations and the accompanying experience of sensory information without a direct external stimulus (Pearson et al., 2015). While the role of memory in decision making has been extensively investigated (Reyna et al., 2003; Weber and Johnson, 2006), research focused on studying specific functions of mental imagery in choices under risk or uncertainty is more scarce (for a review see Zaleskiewicz et al., 2023).

When people consider the future, they can use mental images to envision the possible outcomes of their decisions (Taylor et al., 1998), “pre-experience” how rewarding or threatening the consequences of their choice will be (Blackwell, 2020), and “try-out” various versions of what might happen, depending on which course of action is chosen (Beach, 2009; Ji et al., 2016). For example, people can use their imagery to visually simulate both the form and size of a danger, which allows the subjective severity of risk to be estimated. In such a case, risk perception may be an indirect effect of the liveliness of mental images created by a decision maker (Marks, 1999), with liveliness reflecting “how dynamic, vigorous and alive the image is” (p. 570). In line with that, when people generate lively and negative mental images when faced with a risky situation, they would perceive risk as higher. In contrast, when they produce lively but positive mental images, they could be expected to perceive risk as lower. What is more, when thinking about risky options, people consider not only negative, but also positive outcomes, which means that risk perception might be considered the result of a trade-off between expected losses and benefits (Weber et al., 2002). This suggests that subjective risk evaluation involves the processing of both positive and negative mental images. It should be noted, however, that people tend to underrepresent action errors in their mental imagery compared with the case in which they actually execute an action (Rieger et al., 2011; Dahm and Rieger, 2019). This suggests that in the context of risky decision making, fewer negative outcomes may appear in imagined than in executed risky behaviors.

People use mental imagery not only when being confronted with uncertainty, but also in safe situations in which risk is not involved–for example, most of them could easily imagine spending time with their friends in a nice cafeteria. However, different arguments seem to support the thesis that when people are faced with risk, they use their mental imagery more intensely than when they encounter non-risky situations. First, processing mental images of risk, which, by definition, is a concept related to the possibility of harmful outcomes seems highly adaptive: the capacity for humans to mentally project themselves forward is considered a crucial evolutionary advantage (Suddendorf and Corballis, 1997, 2007; Manuck et al., 2003; Suddendorf and Busby, 2003; Dudai and Carruthers, 2005; Rick and Loewenstein, 2008; Bulley et al., 2020). If people, when faced with a severe threat, can rapidly generate mental images that visually portray scenes of suffering the negative consequences of risk taking, their risk perception may increase and become more accurate (Sinclair et al., 2021), which would protect them against exceeding the limits of acceptable risk. In this sense, mental images might operate similarly to somatic markers defined as changes in the body and brain triggered by one’s perception of specific (e.g., threatening) external events (Bechara and Damasio, 2005).

Importantly, in our view, generating visual mental images goes beyond carefully analyzing future outcomes and their likelihoods, since rational considerations are not only time consuming but also require high risk literacy (Reyna et al., 2009; Cokely et al., 2012). The visual processing that is typically involved in mental imagery is not only faster than verbal thought but can also evoke strong emotional reactions (Blackwell, 2020). When people are faced with the dilemma of how much risk to accept (e.g., whether to continue a risky climb under worsening weather conditions), it is important that the choice is made relatively quickly and that the limits of controlled risk are not exceeded. In such a situation, using mental imagery may not only be useful but also effective. On this basis, we hypothesized that presenting people with risky situations, in comparison to non-risky situations, will evoke a stronger neural response in brain regions that are associated with mental imagery (see section below about neural basis). Such an effect would support our theoretical assumption that mental imagery is especially active when people have to deal with risky situations that can be associated with a threat to their safety and wellbeing.

### Risk perception as a product of mental imagery and emotions

In the present study, we predict that being confronted with risky, in contrast to non-risky situations, will be associated with greater neural activation in brain areas linked both to mental imagery and emotions. The rationale for this prediction is that generating mental visualizations has the capacity to evoke affect (Holmes and Mathews, 2005; Holmes et al., 2008; Blackwell, 2020), which, in turn has an impact on risk perception (Traczyk et al., 2015). This means that risk perception may also depend on the valence of mental imagery in such a way that positive mental images boost pleasant feelings, while negative mental images amplify those that are distressing. An example would be the case of an individual who, when considering their engagement in an exciting but highly dangerous behavior, generates an affect-laden image of a severe accident, resulting in both an intense emotional experience and a rise in the perception of the threat.

As already noted, various models of risk suggest that emotions play an important role in decision making (Loewenstein et al., 2001; Lerner et al., 2015; Zaleskiewicz and Traczyk, 2020), but such models do not always investigate the origins of feelings. Prior research has provided initial evidence to support the theoretical claim that affective evaluation of risk originates in imagery-based processes. For example, it has been shown that the way in which people perceived the risk associated with a nuclear waste repository was strongly related to both affective responses and their imagery of this risk (Slovic et al., 1991). Peters and Slovic (1996) found a significant and positive correlation between affect associated with participants’ mental images related to nuclear power and their support for nuclear power plants. Moreover, more recent research investigated affect and emotions as mediators of the relationship between mental imagery and risk perception. Sobkow et al. (2016) reported that generating mental images of negative outcomes of risk taking, compared to generating images of positive consequences or a neutral condition, boosted negative emotionality and increased risk perception. In the same vein, Zaleskiewicz et al. (2020) revealed that entrepreneurs, compared to controls, produced more positive mental images of potential consequences of their involvement in different risky business projects and, as a result, declared a greater preference for accepting risk. This suggests that the positive mental images they generated decreased their risk perception.

To summarize, existing research demonstrates that mental imagery may be an important psychological factor in risk evaluation. However, all the above-reviewed studies used self-report measures of mental imagery, which have potential limitations, suggesting that the reported findings should be interpreted with caution (see Dahm, 2020 for an extensive review of limitations related to the use of self-report measures of action imagery ability). To address this limitation, in our present study, we used neuroimaging techniques to provide evidence that being faced with risky situations induces a stronger neural response in brain areas involved in mental imagery than when confronted with non-risky situations. Because processing mental images is strongly linked to affect (Holmes and Mathews, 2005; Holmes et al., 2008; Blackwell, 2020), we also predicted more neural activation in areas associated with experiencing emotions as a result of exposure to risky situations (in comparison to non-risky situations).

### Neural basis of mental imagery and its relationship with vividness, emotion, and risk perception

Prior research has demonstrated that mental imagery engages similar brain areas to perception in the same modality (Kosslyn et al., 2001). For example, auditory mental imagery engages the superior temporal gyrus (Aleman et al., 2005; Zvyagintsev et al., 2013), olfactory imagery is associated with activations in the primary olfactory (piriform) cortex (Plailly et al., 2012) and visual mental imagery activates the occipital lobe, including the early visual cortex (Klein et al., 2004). In a recent review on visual mental imagery, Pearson (2019) proposed a top-down general model of voluntary mental imagery based on the sensory representation of information retrieved from memory–a reverse visual hierarchy (see Dentico et al., 2014; Dijkstra et al., 2017 for more details). This model suggests the existence of a large neural network encompassing, among others, frontal areas involved in organizational and executive tasks, medial temporal areas associated with memory retrieval and spatial information, and primary sensory areas implied in visual representation. Interestingly, the levels of activation of several of the brain areas associated with visual mental imagery, such as early visual cortex, precuneus, medial frontal cortex, and the right parietal cortex, have shown to be positively correlated with the experienced vividness (Cui et al., 2007; Dijkstra et al., 2017).

In line with the assumption that mental imagery has the power to elicit emotions (Phan et al., 2002; Holmes and Mathews, 2005; Holmes et al., 2006, 2008; Ji et al., 2016; Blackwell, 2020), mental images might be expected to evoke neural responses in brain areas that are involved in emotional processing. For example, Hoppe et al. (2021) demonstrated that mental images of fearful stimuli, compared with neutral stimuli, were related to increased activation in such regions as the amygdala, insula, mid-cingulate cortex, thalamus and cerebellum. Greening et al. (2022), in a study using mental imagery to generate differential fear conditioning, observed significantly greater activation in the right anterior insula, right dorsolateral prefrontal cortex and bilateral inferior parietal lobe when imagining fear-conditioned stimuli compared with safe-conditioned stimuli.

Finally, for the purpose of the present study, it is important to note that research investigating the neural substrates underlying risky behavior has identified a brain network that comprises numerous areas associated with emotional processing, such as the insula, anterior cingulate cortex, amygdala, thalamus or ventromedial prefrontal cortex (Vorhold, 2008; Mohr et al., 2010; Megías et al., 2015, 2018). Particularly relevant for risk perception is the role of the insula, a brain region commonly related to the processing of aversive emotions (e.g., fear, sadness, or anxiety) that appears to be implied in the estimation of the potential negative consequences associated with risk stimuli (Mohr et al., 2010; Straube and Miltner, 2011; Megías et al., 2018). Nevertheless, to our knowledge, no previous studies have explored the neural basis of risk perception in the context of mental imagery.

### The present study

We propose that when people are confronted with a risky situation and must evaluate the level of threat, they can create a mental visualization of the potential consequences to better understand how they feel about that situation (Traczyk et al., 2015). One reason underlying the expected relationship between mental imagery and risk perception is that generating vivid mental images typically leads to experiencing intense emotions: negative mental images evoke negative affect, and positive mental images evoke positive affect (Holmes and Mathews, 2005, 2010; Holmes et al., 2006, 2008; Blackwell, 2020). Given that mental imagery may induce emotions and that emotions have an impact on risk appraisal, we postulate that: (1) mental imagery could be involved in risk perception; (2) when people are faced with risky situations, they generate and process mental images more intensely than when faced with non-risky situations; and (3) these mental visualizations engage emotions that would be also involved in risk perception.

In the current experiment, we used functional magnetic resonance imaging (fMRI) to register the brain activity of participants who were asked to imagine the consequences of various risky and non-risky situations. To ensure that the experimental manipulation of the task worked correctly, each participant rated the vividness of these mental images and the fear and perceived risk associated with each situation. Additionally, we also controlled individual differences in temperamental emotional reactivity and ability to produce vivid mental images–psychological constructs that might be related to mental imagery and risk perception. At the neural level, we predicted that emotional mental imagery (Blackwell, 2020) in response to risky situations (compared to non-risky situations) would be shown by enhanced activation in those brain regions involved in mental imagery and emotional processing previously described.

## Materials and methods

### Participants

Participants were informed about the fMRI study via an announcement on the GumTree portal. Sixty-six volunteers (*M*_*age*_ = 26.80, *SD*_*age*_ = 4.83, max = 43, min = 20) took part in a screening online questionnaire study, including measures of the vividness of mental imagery (VVIQ; Marks, 1973) and emotional reactivity (ER; Strelau and Zawadzki, 1993). We used a 16-item Polish version of the VVIQ scale (Cronbach’s α = 0.87). The participants were required to rate the vividness of every four items (e.g., “The exact contours of face, head, shoulders and body”) describing four separate scenarios (e.g., “Think of some relative or friend whom you frequently see (but who is not with you at present) and consider carefully the picture that comes into your mind’s eye.” on a 5-point scale (from 1–“No image at all, I only “know” I am thinking of the object” to 5–“Perfectly realistic, as vivid as real seeing”). The ER scale (Cronbach’s α = 0.90) was used to assess the tendency to react intensely to emotionally-laden stimuli. Participants were asked to rate 20 items (e.g., “It is difficult to hurt my feelings”) using a 4-point scale (from 1–“disagree completely” to 4–“fully agree”). There were no outliers in terms of the individual-differences scores. Since the sample size was restricted mainly by our financial resources and time (Lakens, 2022), we sent an email to the pool of 66 participants who completed the screening study, informing them that the first 31 participants who confirmed their interest in participating in the fMRI study would be invited to the neuroimaging laboratory. There were no other exclusion criteria. A total of 31 right-handed volunteers (20 females; *M*_*age*_ = 26.5, *SD*_*age*_ = 6.2) from the community sample were selected for the fMRI study. All participants reported no neurological or psychiatric disorders and gave informed consent before the study. They were informed about the general design of the task and that they could withdraw at any time without any consequences. Each participant received financial compensation of 100 Polish zlotys; PLN (approximately $25). Two participants were excluded from further analyses because of scanner failure and one participant decided to withdraw from the study. The procedure was approved by the ethical committee at SWPS University.

### Materials and procedure

For the experimental task, we used 40 brief descriptions of 20 risky situations (e.g., “you are investing a large amount of money in stocks”) and 20 non-risky situations (e.g., “you are reading a book”; a full list of situations is given in the Supplementary materials). We generated the list of risky situations based on previous studies (e.g., Traczyk et al., 2015; Sobkow et al., 2016) and they covered the five risk domains proposed by Weber et al. (2002). To generate the list of non-risky situations, we asked a group of people to provide examples of situations that were, in their opinion, not linked to risk. The descriptions of risky and non-risky situations were of a similar length (*p* > 0.05). To validate our stimuli (risky and non-risky situations), we asked, in an independent online study, 60 participants (who took part neither in a screening online study nor the fMRI study) to rate the risk associated with each situation (1–“not risky at all”; 5–“extremely risky”), the valence of feelings evoked by the situation (−2–“negative”; 2–“positive”), and ease of imagining the situation (1–“very easy”; 5–“extremely difficult”). We found that risky situations, in comparison with non-risky situations, were rated as more risky (*p* < 0.001), more negative (*p* < 0.001), and more difficult to imagine (*p* < 0.001). Based on these results, we decided to consistently use the terms “risky situations” and “non-risky situations” throughout the whole manuscript.

**FIGURE 1:**
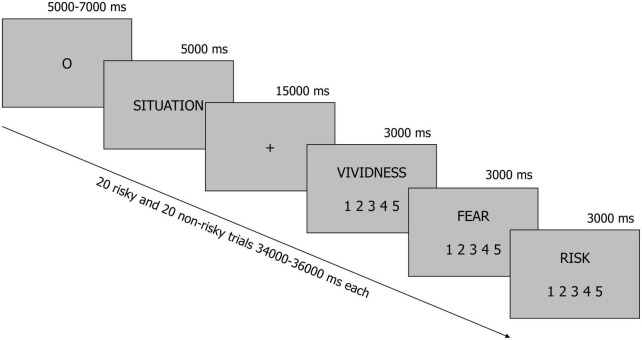
A schematic illustration of the experimental procedure. Participants were presented with brief descriptions of 20 risky and 20 non-risky situations. In each trial, they were instructed to imagine all consequences of a situation for 15 s when a fixation cross was displayed on the screen. After this, they rated vividness, fear and perceived risk.

All situations were presented to participants in the MRI scanner in black font on a gray background (Figure 1). Each trial started with an oval fixation point (presented for a pseudorandomly chosen period of time ranging from 5 to 7 s–the fixation time was constant across participants and situations), which was immediately followed by a description of the situation displayed for 5 s. Next, participants were instructed to imagine all consequences of the presented situation for 15 s when a fixation cross was presented on the screen. Finally, participants used three 5-point scales to rate vividness (1–“not vivid at all,” 5–“very vivid”), fear (1–“not at all,” 5–“very much”), and perceived risk (1–“not risky at all,” 5–“very risky”) that were associated with each situation. Of the many emotions that people can experience when faced with risk, we focused on fear, because this is the feeling people most typically refer to when forming their risk perceptions (Slovic, 1987; Sjoberg, 2002). All questions were presented in a fixed order, whereas the situations were arranged in a pseudorandom order.

All materials were presented to participants in the Polish language because the experiment was conducted in Poland.

### fMRI data acquisition

Structural and functional magnetic resonance images were acquired using a Siemens 3-Tesla Trio MRI scanner with a 32-channel head coil at the Laboratory of Brain Imaging, Nencki Institute of Experimental Biology (Warsaw, Poland). Before the main fMRI experiment, participants completed a training session in a mock scanner where they were familiarized with the equipment, study conditions, and modes of responses. Participants were instructed to remain relaxed and motionless during the scan. In addition, foam pads were used to limit head motions and reduce scanning noise. Participants were only allowed to move the right index finger to make their responses during the task by pressing a response-box button.

T1-weighted images were obtained using a magnetization-prepared rapid gradient-echo sequence (MPRAGE) with a repetition time (TR) of 2,530 ms, an echo time (TE) of 3.32 ms, and a flip angle of 7°. For each volume, 176 axial slices of 1 mm thickness were acquired, which allowed the whole brain to be covered with the following parameters: voxel size = 1 mm× 1 mm× 1 mm, matrix size = 256 × 256 voxels and FOV = 256 mm. Functional images were obtained using a T2*-weighted echo-planar sequence with a TR of 2,000 ms, TE of 25 ms, and a flip angle of 90°. Each volume, covering the whole brain, consisted of 39 axial slices parallel to AC-PC plane with 3.5 mm thickness each: voxel size = 3.5 mm× 3.5 mm× 3.5 mm, matrix size = 64 × 64 voxels and field of view (FOV) = 224 mm.

### fMRI pre-processing

First, all anatomical and functional images were reoriented to the anterior commissure. For each participant, functional volumes were motion-corrected via spatial realignment to the mean image after a previous realignment to the first volume and co-registered with the individual structural T1-weighted image. Next, these images were spatially normalized to the standard Montreal Neurological Institute (MNI) space and resampled to a resolution of 3 × 3 × 3 mm. Finally, they were smoothed by Gaussian kernel (8 mm full width at half-maximum).

In order to evaluate levels of head motion, we computed the index of framewise displacement (FD) for each participant from the 6 translational and rotational motion parameters by Power’s method (Power et al., 2015). The mean FD was 0.14 ± 0.04 mm (ranging from 0.07 to 0.28 mm), indicating that head motion was low in all the sample. No participants were excluded because of excessive head motion following criteria usually employed in previous fMRI literature: mean FD > 0.3 mm and > 20% of the volumes above FD > 0.3 [note that these criteria are common in resting-state fMRI, which are usually far more conservative than those employed in task-based fMRI (Power et al., 2015; Achterberg and van der Meulen, 2019; DeSerisy et al., 2020; Zhang et al., 2020; Narita et al., 2021)].

### fMRI data analysis

Statistical analyses of fMRI data were restricted to comparisons between risky and non-risky situations. Neural correlates associated with the rating scales scores were not included in the analyses since the experimental task was designed to identify neural differences between risky and non-risky conditions. The main aim of the rating scales was to check that the experimental manipulation was successful; therefore, there was not an appropriate control of the rating scores that allowed us to properly perform a trial-level analysis that includes these variables.

We adopted a two-level general linear model approach. In the subject-specific first-level model, experimental conditions (risky situations and non-risky situations) were convolved with the canonical hemodynamic response function. fMRI data for each condition were time-locked to the onset of the reading phase with a duration of 20 s (until the end of the imagery phase). Serial autocorrelations were corrected using an autoregressive (AR) 1 model, with a high-pass filter (128 s) to reduce low-frequency noise. We computed two whole-brain contrasts in order to determine brain areas showing differences between conditions: risky situations > non-risky situations and risky situations < non-risky situations.

The resulting contrast images from each participant’s first-level analysis were entered into the second-level (group) analysis. A one-sample *t*-test was performed to determine significant activation at the group level. We adopted a non-parametric cluster-based permutation approach using the SnPM13 toolbox integrated within the SPM toolbox (Statistical non-Parametric Mapping)^1^ (Nichols and Holmes, 2002). Cluster-based permutation tests were used to control for multiple comparisons due to their better fit to the spatially correlated nature of the fMRI signal and their higher sensitivity to weak and diffuse changes in the BOLD signal, particularly with moderate sample sizes (Heller et al., 2006; Woo et al., 2014). The number of permutations was set to 5,000 and the level of significance was *p* < 0.05; this value was family-wise error (FWE)-corrected (cluster-wise *p*-value) using a cluster-forming threshold of *p* < 0.0001 (voxel-wise *p*-value). Given the previous reports in the literature showing gender and age differences in risk behavior (Weber et al., 2002; Steinberg, 2010; Sánchez-López et al., 2022), we decided to introduce gender and age as covariates of non-interest in the analysis.

In addition, as a secondary aim, we were interested in exploring how the possible differences in brain activation found in the previous risky versus non-risky contrast (at the whole trial level) can vary throughout processing of the task (a time-course analysis). To this end, the temporal sequence of the trial was divided into four 5-s bins during the first-level (subject-specific) analysis. We employed 5-s bins to align the duration of the imagery phase (15 s) with that of the reading phase (5 s). In other words, the imagery phase was divided into three equally-long phases. fMRI data for the first bin were time-locked to the onset of the reading phase and data for the second, third, and fourth bins were time-locked to 5, 10, and 15 s, respectively, after onset of the reading phase (i.e., the second bin was time-locked to the onset of the imagery phase). Analysis was restricted to a set of regions of interest defined from the significant clusters found in the whole trial (using an implicit mask). In this case, given that the analysis was not carried out across the whole brain, we decided to adopt a non-parametric voxel-based permutation approach to conduct the second-level (group) analysis (SnPM13 toolbox; 5,000 permutations; *p* < 0.05, FWE corrected; Nichols and Holmes, 2002). Gender and age were included as covariates.

Image pre-processing and statistical analyses were conducted in SPM12 (Wellcome Trust Centre for Neuroimaging, University College London, UK).^2^ Brain regions were identified by automated anatomical labeling 3 atlas (AAL3; Rolls et al., 2020). We declare that all methods were carried out in accordance with relevant guidelines and regulations.

## Results

### Behavioral results

The descriptive statistics and correlations between self-report measures are presented in Table 1 (Supplementary Tables S1, S2 contain descriptive statistics and correlations for each condition, separately). We found that higher risk perception was related to higher ratings of fear (*r* = 0.816, *p* < 0.001) and higher scores on the ER scale (*r* = 0.388, *p* = 0.041). Ratings of the vividness of presented situations were positively related to the ability to create vivid images, as measured by the VVIQ (*r* = 0.457, *p* = 0.015). Additionally, the correlation between fear ratings and ER scores was significant (*r* = 0.583, *p* < 0.001), suggesting that the measures used in the fMRI procedure were valid.

**TABLE 1:** Descriptive statistics and Pearson’s *r* correlation coefficients.

Variable	*M*	*SD*	Risk	Fear	Vividness	VVIQ
1. Risk	2.38	0.36	–			
2. Fear	2.25	0.38	0.816***	–		
3. Vividness	4.06	0.49	–0.095	0.019		–
4. VVIQ	63.04	8.29	–0.175	–0.283	0.457*	–
5. ER	39.29	8.35	0.388*	0.583***	–0.187	–0.487**

ER, emotional reactivity; VVIQ, Vividness of Visual Imagery Questionnaire; Risk, fear and vividness refer to ratings of situations provided by participants in the scanner. *p < 0.05, **p < 0.01, and ***p < 0.001.

Next, we conducted a paired-samples *t*-test to investigate differences in mean ratings of risk perception, fear and vividness between risky and non-risky conditions. We found that ratings of risk perception were higher in the risky condition (*M* = 3.59, *SD* = 0.58) compared to the non-risky condition (*M* = 1.18, *SD* = 0.58): *t*(27) = −24.84, *p* < 0.001 and Cohen’s *d* = −4.69. Fear ratings were also higher in the risky condition (*M* = 3.31, *SD* = 0.64) than in the non-risky condition (*M* = 1.19, *SD* = 0.22): *t*(27) = −19.61, *p* < 0.001 and Cohen’s *d* = −3.71. Interestingly, participants rated their mental images of non-risky situations as more vivid (*M* = 4.39, *SD* = 0.54) than mental images of risky situations (*M* = 3.73, *SD* = 0.56): *t*(27) = 6.71, *p* < 0.001 and Cohen’s *d* = 1.27.

Finally, to address possible non-independence arising from the hierarchical structure of our data (i.e., ratings of risky situations nested in participants), we fitted a hierarchical linear regression model with varying intercepts for participants and situations and varying slopes for the effects of the condition, fear, and vividness on risk perception (the outcome variable). Additionally, we included the VVIQ and ER scores as predictors. All predictors were mean-centered. The non-risky condition was coded as −0.5 and the risky condition as 0.5. The model was estimated in the lme4 package (Bates et al., 2014) and implemented in the R statistical environment (R Core Team, 2020).

Our experimental manipulation was effective. We found that the ratings of risk were higher in the risky condition than in the non-risky condition (*b* = 1.38, *p* < 0.001). Moreover, higher ratings of risk were associated with greater fear (*b* = 0.68, *p* < 0.001) and a greater ability to create vivid visual images as measured by the VVIQ (*b* = 0.09, *p* = 0.024). We did not find a significant relationship between the ratings of vividness and risk perception (*b* = −0.05, *p* = 0.075) or ER and risk perception (*b* = 0.05, *p* = 0.144). It suggests that controlling for individual differences in ER and ratings of fear in the regression model indicates a more robust effect of the latter variable. The fixed and random effects explained *R*^2^ = 0.81 of the variance. Adding gender and age to the model as covariates did not significantly change estimates and the pattern of relationships.

To summarize, we demonstrated that our behavioral task was valid. The situations in the risky condition were indeed rated as riskier in comparison to the non-risky condition. The higher ratings of risk were associated with higher reported fear, showing that risky situations have a greater capacity to evoke strong emotional responses than non-risky situations. We did not find a relationship between risk ratings and vividness; the ratings of vividness were higher for non-risky than risky situations. We provide a potential explanation for this surprising effect in the section “General discussion.”

### Neuroimaging results

**FIGURE 2:**
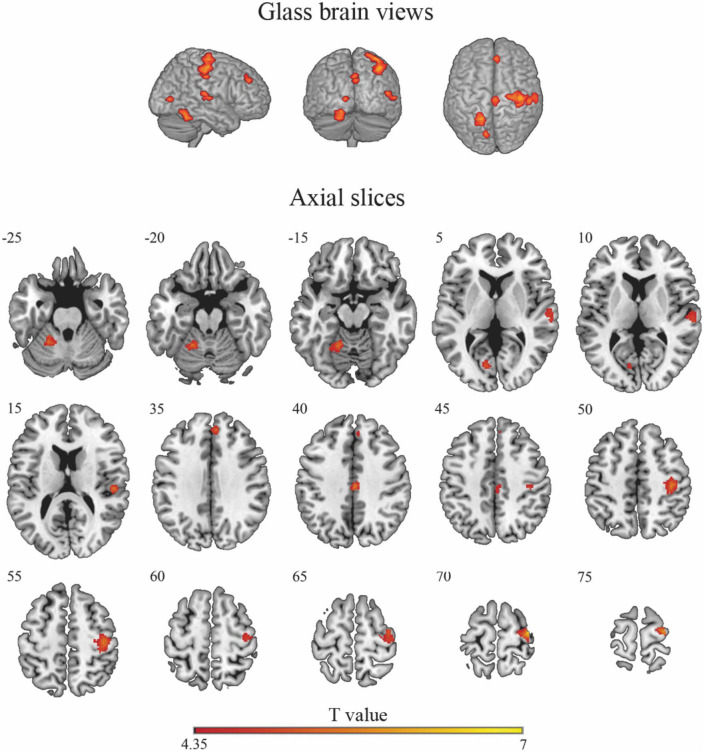
Glass-brain views **(top panel)** and axial maps **(bottom panel)** displaying brain areas with a statistically significant increased BOLD signal for the risky > non-risky contrast. Note that glass-brain images show projections of the activations across the whole brain volume onto two-dimensional axial, sagittal, and coronal views.

Cluster-based permutation analysis of the risky versus non-risky contrasts for the whole period of the trial revealed six significant clusters showing increased BOLD signal activation in the risky situations compared to the non-risky situations (see Table 2; *T* > 4.35, minimum cluster size [k] > 20 voxels). The risky < non-risky contrast revealed no significant differences. The locations of the activation peaks of the significant clusters for the risky > non-risky situations are presented in Table 2. For the right hemisphere, clusters encompassed part of the precentral gyrus, the cingulate gyrus (mid-cingulate area), the medial part of the superior frontal gyrus, and the superior temporal gyrus extending to the insular cortex. For the left hemisphere, clusters included the cerebellum anterior and posterior lobe (activation peak in the anterior lobe) and the calcarine sulcus extending to the cuneus. Figure 2 shows the anatomical localization of the clusters.

**TABLE 2:** Statistically significant clusters (and their peaks) showing greater activation in the risky condition than in the non-risky condition.

Cluster (size)	Brain region	L/R	MNI coordinates			*T*
			***x***	***y***	***z***	
Cluster 1 (*k* = 558)	Precentral gyrus	R	36	−14	68	6.23
Cluster 2 (*k* = 68)	Cingulate gyrus (mid-cingulate area)	R	2	−22	40	5.31
Cluster 3 (*k* = 236)	Cerebellum anterior lobe	L	−18	−50	−24	5.31
Cluster 4 (*k* = 123)	Superior temporal gyrus	R	52	−24	14	5.30
Cluster 5 (*k* = 67)	Superior frontal gyrus (medial)	R	6	42	34	5.22
Cluster 6 (*k* = 47)	Occipital lobe (calcarine)	L	−12	−76	4	5.08

**FIGURE 3:**
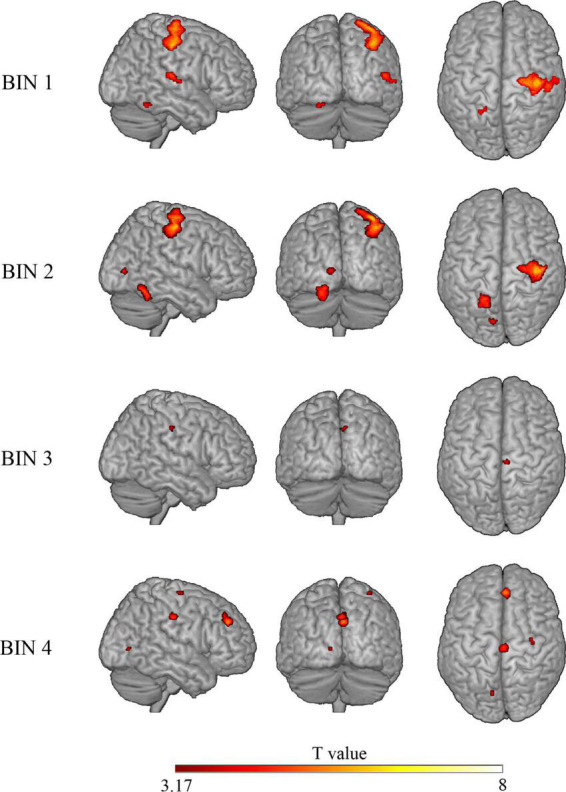
Glass-brain views (sagittal, coronal, and axial) displaying brain areas with a statistically significant increased BOLD signal in the risky > control contrast for each of the four bins. Analysis is restricted to clusters showing significant differences in the “risky > control” contrast on the whole trial.

To study the processing of risk in more detail, we decided to explore the brain activation time-course in the significant clusters observed in the risky > non-risky contrast, dividing each trial into four bins of 5 s (see ‘Materials and methods’ section). Results for Bin 1 revealed three clusters (*T* > 3.17) involving the postcentral gyrus, precentral gyrus, cerebellum anterior lobe, and superior temporal gyrus extending to Rolandic operculum and insular cortex. Bin 2 showed differences in three clusters involving the precentral gyrus, occipital lobe, and cerebellum posterior lobe (*T* > 3.48). Bin 3 showed one cluster in the cingulate gyrus (mid-cingulate area) (*T* > 3.58). Finally, Bin 4 showed four clusters involving the medial part of the superior frontal gyrus, cingulate gyrus (mid-cingulate area), precentral gyrus, and occipital lobe (*T* > 3.39). Table 3 provides details of the significant clusters for each bin. Figure 3 presents the anatomical localization of the cluster found in each bin through a series of sagittal glass-brain projections.

**TABLE 3:** Statistically significant clusters (and their peaks) showing greater activation in the risky condition than in the non-risky condition for each of the four bins into which the trials were divided.

Cluster (size)	Brain region	L/R	MNI coordinates			*T*
			***x***	***y***	***z***	
**Bin 1–reading (5 s)**						
Cluster 1 (*k* = 543)	Postcentral gyrus	R	40	−20	48	7.19
	Precentral gyrus	R	32	−18	70	6.25
Cluster 2 (*k* = 83)	Rolandic operculum	R	52	−22	16	4.46
	Superior temporal gyrus	R	60	−12	8	3.67
Cluster 3 (*k* = 14)	Cerebellum posterior lobe	L	−26	−52	−20	3.45
	Cerebellum anterior lobe	L	−18	−46	−20	3.28
**Bin 2–imagery (first 5 s)**						
Cluster 1 (*k* = 546)	Precentral gyrus	R	38	−14	66	7.26
Cluster 2 (*k* = 153)	Cerebellum posterior lobe	L	−22	−54	−20	5.81
Cluster 3 (*k* = 24)	Occipital lobe (Calcarine)	L	−12	−78	4	4.18
**Bin 3–imagery (from 5 to 10 s)**						
Cluster 1 (*k* = 12)	Cingulate gyrus (mid-cingulate area)	R	8	−22	42	4.29
**Bin 4–imagery (last 5 s)**						
Cluster 1 (*k* = 68)	Superior frontal gyrus (medial)	R	0	42	32	6.39
Cluster 2 (*k* = 45)	Cingulate gyrus (mid-cingulate area)	R	2	−22	38	5.11
Cluster 3 (*k* = 12)	Precentral gyrus	L	36	−14	68	3.92
Cluster 4 (*k* = 8)	Occipital lobe (Lingual gyrus)	L	−10	−72	2	3.83

The analysis was restricted to regions of interest defined from the significant clusters found in the previous risky > non-risky analysis performed for the whole trial.

To summarize, findings from the neuroimaging component of the study supported our predictions. In particular, we demonstrated that mental images of risky situations (compared with mental images of non-risky situations) were related to higher neural activation in brain areas that are usually involved in mental imagery, emotions, and the processing of risk.

### General discussion

The main aim of the present research was to empirically investigate the theoretical idea that when people are confronted with risky situations, they tend to both generate visual mental images and experience emotions to a greater extent than when they face non-risky situations. Unlike in previous studies that tested similar predictions but used self-report measures of mental imagery, here we used a neuroimaging technique (fMRI) to verify the hypothesis that visualizing risky situations induces a stronger neural response in brain areas associated with mental imagery and emotions than visualizing non-risky situations.

We identified several brain regions that were more strongly activated when participants produced mental images of risky situations compared with non-risky situations. In particular, we found that these regions largely overlap with brain areas that previous literature have linked, among other functions, to visual mental imagery (such as the occipital lobe; Kosslyn et al., 2001; Ganis et al., 2004; Pearson, 2019; Bartolomeo et al., 2020), speech imagery (superior temporal gyrus; Aleman et al., 2005) and movement imagery (medial frontal gyrus, precentral gyrus, and cerebellum; Klein et al., 2004; Fulford et al., 2018; Winlove et al., 2018). These findings seem to support our prediction that visualizing risky as opposed to non-risky situations would result in intensified activation of the brain areas associated with mental imagery. Additionally, using self-report measures, we observed that the vividness of the generated mental images correlated positively with scores on the VVIQ which measures individual differences in people’s ability to produce vivid visual mental imagery. This indicates that some people may be prone to more intensely visualizing the potential consequences of risky situations. This result was corroborated by correlations among self-report measures conducted separately for risky and non-risky situations (see Supplementary materials). For both categories of situations, this correlation was positive and significant, indicating that people’s abilities to generate vivid mental images are important not only in non-risky situations (such as those that are used in this questionnaire) but also in situations associated with risk.

Interestingly, we found that producing mental images of risky, as opposed to non-risky, situations was associated with increased neural activation in the cerebellum and insular cortex–both regions implicated in emotional processing. This finding is in line with growing evidence showing that cerebellum, apart from motor functions, is also involved in the processing of emotions, particularly the cerebellum posterior lobe (Stoodley and Schmahmann, 2010; Baumann and Mattingley, 2012; Adamaszek et al., 2017). As for the insula, prior research has demonstrated that the activation of this brain area is associated with emotional recall/imagery (Phan et al., 2002; Hoppe et al., 2021; Greening et al., 2022) and that increased fear appears as a typical response to risk (Slovic, 1987; Marris et al., 1997; Sobkow et al., 2020). Moreover, the insula has shown to play a central role in estimating the potential negative consequences of the risk-taking behavior (Mohr et al., 2010; Megías et al., 2018). At this point it is necessary to note that, while prior literature has shown the involvement of different subregions of the insula in emotion and risk-taking (Straube and Miltner, 2011; Reske et al., 2015; Centanni et al., 2021; Greening et al., 2022), the anterior insula has been particularly highlighted. In our case, activation in risky conditions, compared to non-risky conditions, mainly encompassed portions of the posterior insula. Further research studying insula subregions with more precision is needed to understand better the insula contribution (see Uddin et al., 2017).

Supporting the greater activation of brain regions associated with emotional processing during the risky situations, we observed two clear tendencies at the behavioral level. First, and supporting previous research (Traczyk et al., 2015; Sobkow et al., 2016), our study indicated that risk perception was intensified when people experienced stronger fear as a consequence of generating visual mental images of risk. This result might also suggest that the emotional response evoked due to participants’ exposure to risky situations and observed on the neural level was fear. Second, this effect was confirmed by the correlation between risk perception and dispositional ER (defined in terms of the tendency to experience frequent and intense emotional arousal); those participants who declared that they are more emotionally reactive estimated the risk as higher.

From the perspective of mental imagery and risk perception, these findings are of special importance because activation in insula and cerebellum could be elicited by the mental image of a fear-related stimulus in the absence of an actual percept (Hoppe et al., 2021). Consequently, this means that emotional response to risk might be evoked by the mental image of risk itself, and a decision maker does not have to face the real risk to generate an adaptive course of action.

Finally, when participants were faced with risky situations, we also observed an increased BOLD signal in the cingulate gyrus (mid-cingulate area), which is usually recruited in reward-based decision making and monitoring of decision outcomes. In particular, this area exhibits increased activity when people process information about a decision, make predictions, and monitor possible outcomes and consequences (Alexander and Brown, 2011; Apps et al., 2013; Silvetti et al., 2014). Mental imagery of risk allows for the different consequences and outcomes of a risky action to be simulated without experiencing them directly. Such emotionally laden mental images of outcomes are processed and integrated in order to estimate the riskiness of different alternatives preceding subjective selection of the one that is considered optimal.

The exploratory time-course analysis in four bins seems to support these temporal dynamics; neuroimaging data suggested that at the beginning of a trial (i.e., when participants started processing a risky situation) there was an enhanced activation in areas associated with mental imagery and emotions. Along with the subsequent processing of the risky situations, significant activation in the cingulate gyrus emerged, suggesting the engagement of higher-order cognitive functions related to making predictions, monitoring outcomes, and decision making. These results are in line with neural models of mental imagery (Pearson et al., 2015) and risky decision making (Vorhold, 2008; Mohr et al., 2010) supporting the involvement of primary sensory areas in visual imagery representation and indicating that mental images of risk-related situations can elicit emotional neural responses associated with potential losses, which prepare the individual to make decisions that help to avoid the unwanted outcomes. In any case, it is important to note that some of the brain regions identified in this study could also be related to other cognitive functions, thus, in order to give stronger support to our inferences, future studies should focus on more specific characteristics of the task which allows to exclude other possible interpretations. Moreover, it is important to note the limitations of fMRI and the study of the hemodynamic response in terms of temporal resolution and time-course analysis. The use of other neuroimaging techniques such as functional near-infrared spectroscopy (fNIRS) or the combination of electroencephalography (EEG) and fMRI could help to confirm the dynamics of mental imagery processes.

Building a theoretical model explaining the interplay between mental imagery, emotions, risk perception, and decision making is undoubtedly a challenge for future research. Although several notable theoretical models (Loewenstein et al., 2001; Lerner et al., 2015) posit that judgment under risk and uncertainty can be shaped by both anticipated and experienced emotions, none of these frameworks implemented the construct of emotional mental imagery (Blackwell, 2020) in decision making (Zaleskiewicz et al., 2023). In the present study, we have provided initial evidence that mental imagery–along with emotional responses–might serve as an input to risk perception. Nevertheless, there are still some open questions that should be addressed. First, the results of the present study indicated that exposure to risk evokes a more intense neural response related to mental imagery and emotions than exposure to non-risky stimuli, but they do not allow us to draw conclusions about the nature of the relationship between emotions and mental imagery in risk perception. It seems that both causal links (i.e., mental imagery evokes an emotional response) and reciprocal links (i.e., the emotional response produced by mental imagery becomes a basis for new mental images that differ in content, valence, or vividness) might be considered and investigated in future research.

Second, the tendency to either accept or reject risk may be moderated by both the valence of mental images and their vividness. When people are faced with the prospect of risky decision making and are free to generate images of its consequences, they may visualize not only negative outcomes (threats) but also positive outcomes (benefits). For example, imagining possible outcomes of risky investments on the stock market may result in either positive visualizations (such as earning money and consequently meeting various needs) or negative visualizations (such as losing money and getting into serious financial trouble), creating a risk/reward tradeoff. It can be also assumed that the effect of the negative versus positive mental imagery on risk perception might be intensified by the vividness factor. In the present study, we did not find correlations between self-report measures of the vividness of mental imagery and risk perception and observed that participants reported greater vividness of their mental images of non-risky than risky situations. In our view, these results might be driven by the lack of control over the valence of mental images in our experimental design–participants were asked to produce mental images and report on their vividness but not to rate the degree to which these images were either positive or negative. Because we consider the lack of valence measurement as a limitation of our study, we suggest that future research investigating the psychological functions of mental imagery on risk perception should focus not only on the predictive power of vividness but also on the effect of the interaction between vividness and valence.

Third, it is important to note that the concept of vividness itself can be understood as a combination of clarity and liveliness (Marks, 1973, 1999; McKelvie, 1995), where clarity reflects the detail of the mental image (plus the brightness of its colors and the sharpness of the outline) while liveliness refers to the extent to which an image is dynamic, vigorous, and alive. Given the relevance of the emotional component in risk perception, the strongest neural response that we have observed in brain areas associated with mental imagery and emotions could be related to liveliness (i.e., the similarity in intensity between imagery and real performance) rather than the clarity and detail of the mental images generated by participants. This might also explain why, at the behavioral level, our participants rated the vividness of non-risky situations higher than that of risky situations. It is possible that the non-risky situations that we presented to participants in the present study were more common and everyday than the risky situations, and therefore mental images related to them were also clearer and more detailed than those generated in response to risk. The results of other research have shown that people provide higher vividness ratings for visual mental imagery of familiar stimuli in comparison to unfamiliar stimuli (Ragni et al., 2021). In other words, the effect we observed in our study at the neural level could potentially be a consequence of the liveliness aspect of mental imagery whereas the effect found at the self-report level was more concerned with clarity. However, this explanation requires further empirical investigation.

Fourth, prior evidence has shown a close association between mental imagery and episodic memory (Kosslyn et al., 2001; Schacter et al., 2008). It means that people can base the generation of mental images related to risk on their past experiences, and the valence of mental imagery might be determined by the negativity/positivity of the memories they have. Future research applying the fMRI method could be useful in disentangling the specific role of episodic hindsight and episodic foresight in risk perception, contributing in a new manner to the ongoing debate about the functions of memory in decision making under risk (Weber and Johnson, 2006).

Fifth, the present study was designed as a within-subject experiment, in which participants were presented with two types of situations (risky vs. non-risky), but all of them were instructed to generate mental images. In future studies, it would be useful to have another condition, in which participants do not receive instructions to engage in producing mental images. This could allow us to investigate whether people faced with risk process mental imagery spontaneously without being encouraged to do so. Moreover, another point to consider is that, given the prolonged duration of the imagery phase (15 s), participants could be susceptible to resting state and mind wandering during this phase. Future studies should examine whether participants imagine each situation for the full duration of the imagery phase, by, for example, using post-scan manipulation check questions.

Finally, due to the limited sample size in the present study, it is recommended that future research be conducted with a larger group of participants to replicate the effects reported.

To conclude, we have demonstrated that mental images of risky situations, as opposed to non-risky situations, are associated with increased neural activation in brain areas that have been traditionally linked to mental imagery processes, emotional processing, and decision making. Our findings suggest that the evaluation of everyday risky situations may begin with visualizing the potential consequences of risk and may be driven by emotional responses (e.g., fear) that result from dynamic, alive, and vigorous mental imagery.

## Data availability statement

The behavioral data used and/or analyzed during the current study are available at https://osf.io/pbjh2/; neuroimaging data are available from the corresponding author upon reasonable request.

## Ethics statement

The studies involving humans were approved by the Ethics Committee at the SWPS University. The studies were conducted in accordance with the local legislation and institutional requirements. The participants provided their written informed consent to participate in this study. Written informed consent was obtained from the individual(s) for the publication of any potentially identifiable images or data included in this article.

## Author contributions

TZ, JT, and AS theoretical model. TZ, JT, AS, and KF methods and conducting research. JT, AS, KF, and AM-R data analysis. All authors contributed to writing the manuscript and approved the submitted version.

## Footnotes

1
http://warwick.ac.uk/snpm

2
http://www.fil.ion.ucl.ac.uk/spm/
