# Supplementary material for: Visualizing risky situations induces a stronger neural response in brain areas associated with mental imagery and emotions than visualizing non-risky situations
Source: Front Hum Neurosci. 2023 Sep 19;17:1207364. doi: 10.3389/fnhum.2023.1207364 (PMC10546025; doi:10.3389/fnhum.2023.1207364)
Supplement: Supplementary file 1 [file Data_Sheet_1.docx]

**Visualizing risky situations induces a stronger neural response in brain areas associated with mental imagery and emotions than visualizing non-risky situations**

**Supplementary Materials**

**Brief descriptions of risky (upper list) and non-risky (bottom list) situations used in the experiment**

you are having unprotected sex

you are forging your signature on an important document

you are betting on the weekly earnings in playing cards

you are downloading a work from the internet and presenting it as yours

you are expressing controversial views in a group of people you do not know

you are jumping with a parachute

you are ignoring persistent health problems

you are spreading negative and false information about someone you dislike

you are driving your car fast and dangerously

you are reprimanding your boss or teacher publicly

you are lending a few thousand dollars to your friend

you are travelling alone with a tent in wild, unpopulated places

you are investing a large amount of money on the stock market

you are cutting in line

you are taking hard drugs

you are taking a large mortgage

you are taking strong medications without consulting your doctor

you are inviting a newly met person from work or school on a date

you are kayaking on a rushing mountain river

you are cheating during the exam

you are receiving a book award in a competition

you are getting a compliment from your partner

you are watching an interesting movie in the cinema

you are spending a great weekend out of town

you are reading an interesting article in the press

you are eating a tasty meal

you are successfully passing a difficult exam on the first term

you are reaching your destination safely after a long journey

you are reading an interesting book

you are resting with your friends

you are walking in the park on a sunny spring day

you are visiting a close family member

you are listening to a great album from your favorite band

you are going on a desired vacation during your vacation

you are having a tasty dinner with your friends

you are doing small shopping in a local store

you are getting a discount coupon

you are buying a cinema ticket for a movie premiere

you are getting a significant pay raise at work

you are finishing an important project

Table S1. Descriptive statistics and Pearson’s *r* correlation coefficient for risky situations.

| **Variable** | ***M*** | ***SD*** | **Risk** | | **Fear** | | **Vividness** | | **VVIQ** | |
| --- | --- | --- | --- | --- | --- | --- | --- | --- | --- | --- |
| 1. Risk | 3.59 | 0.58 | – |  |  |  |  |  |  |  |
| 2. Fear | 3.31 | 0.64 | .78 | ** | – |  |  |  |  |  |
| 3. Vividness | 3.73 | 0.56 | -.23 |  | -.05 |  | – |  |  |  |
| 4. VVIQ | 63.04 | 8.29 | -.32 |  | -.34 |  | .30 | * | – |  |
| 5. ER | 39.29 | 8.35 | .43 | * | .65 | ** | -.20 |  | −.49 | ** |

*Note:* ER, emotional reactivity; VVIQ, Vividness of Visual Imagery Questionnaire; Risk, Fear and Vividness refer to ratings of scenarios provided by participants in the scanner.

* *p* < .05, ** *p* < .01, *p* < .001.

Table S2. Descriptive statistics and Pearson’s *r* correlation coefficient for non-risky situations.

| **Variable** | ***M*** | ***SD*** | **Risk** | | **Fear** | | **Vividness** | | **VVIQ** | |
| --- | --- | --- | --- | --- | --- | --- | --- | --- | --- | --- |
| 1. Risk | 1.18 | 0.24 | – |  |  |  |  |  |  |  |
| 2. Fear | 1.20 | 0.22 | .74 | ** | – |  |  |  |  |  |
| 3. Vividness | 4.39 | 0.54 | .18 |  | -.04 |  | – |  |  |  |
| 4. VVIQ | 63.04 | 8.29 | .25 |  | .01 |  | .51 | ** | – |  |
| 5. ER | 39.29 | 8.35 | .14 |  | .16 | ** | -.13 |  | −.49 | ** |

*Note:* ER, emotional reactivity; VVIQ, Vividness of Visual Imagery Questionnaire; Risk, Fear and Vividness refer to ratings of scenarios provided by participants in the scanner.

* *p* < .05, ** *p* < .01, *p* < .001.
